# Interleukin-27 is a multitarget regulator of fibroblast remodeling in thyroid-associated ophthalmopathy

**DOI:** 10.1016/j.isci.2025.113982

**Published:** 2025-11-10

**Authors:** Pengbo Zhang, Xiaofang Wang, Nanji Lu, Yan Nie, Xibo Zhang, Longqian Liu

**Affiliations:** 1 Department of Ophthalmology, West China Hospital, Sichuan University, Chengdu, China; 2 Laboratory of Optometry and Vision Sciences, West China Hospital, Sichuan University, Chengdu, China; 3 State Key Laboratory of Southwestern Chinese Medicine Resources, School of Pharmacy, Chengdu University of Traditional Chinese Medicine, Chengdu, China; 4 Department of Ophthalmology, Affiliated Hospital of Southwest Medical University, Luzhou 646000, Sichuan, China

**Keywords:** fibrosis, immune response, Ophthalmology

## Abstract

Thyroid-associated ophthalmopathy (TAO) is characterized by inflammation and tissue remodeling, including fibrosis and adipogenesis. Here, we identify interleukin-27 (IL-27) as a negative feedback immunomodulator in TAO. Serum IL-27α levels were significantly elevated in patients with TAO compared with healthy and inflammatory disease controls. In orbital fibroblasts (OFs), exogenous IL-27 suppressed IL-1β-induced proinflammatory cytokines and reduced hypoxia-induced NLRP3 inflammasome activation. IL-27 also attenuated TGF-β-driven fibrosis via p38 MAPK signaling in CD90^+^ OFs. Furthermore, in CD90^−^ OFs, IL-27 restrained adipogenic differentiation by dampening AKT signaling and suppressing hypoxia-induced adipogenesis in a ROS-dependent manner. Our results indicate that IL-27 signaling acts as a multifunctional regulator and negative feedback modulator that helps limit the pathological progression of TAO.

## Highlights

• IL-27α is elevated in TAO and correlates with disease activity
• IL-27 dampens inflammation and NLRP3 inflammasome activation in orbital fibroblasts
• IL-27 suppresses TGFβ-induced fibrosis via p38 MAPK in CD90^+^ orbital fibroblast
• IL-27 limits adipogenesis in CD90^−^ fibroblasts by inhibiting IGF-AKT and reducing ROS

## Graphical abstract

**Figure:**
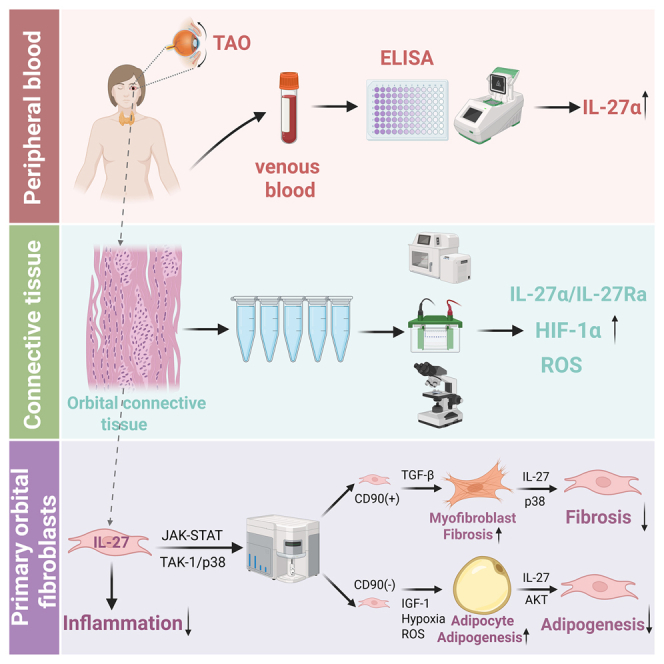

## Introduction

Thyroid-associated ophthalmopathy (TAO), also called Graves’ orbitopathy (GO), is an autoimmune inflammatory condition that constitutes the most frequent extrathyroidal expression of Graves’ disease (GD).^1^^,^^2^^,^^3^ TAO affects the orbital and ocular adnexa, resulting in exophthalmos, eyelid retraction, strabismus, conjunctival injection, corneal ulceration, and even vision loss.^3^ The incidence of TAO is approximately 2/10000 in the population per year and about 25%–40% in patients with GD, which may cause a significant socio-economic burden.^4^ Glucocorticoids are the mainstay of treatment for active TAO, and orbital irradiation is usually used as a supplemental therapy.^5^ Surgery is suitable for the stationary phase to restore normal orbital anatomy and relieve the risk of vision impairment.^6^ However, these approaches often have significant side effects, limited efficacy, and a high relapse rate. Recently, targeted biologics such as teprotumumab (an IGF-1R monoclonal antibody) have shown promise in clinical trials, reflecting a shift toward more specific immune-modulatory strategies.^7^ However, no therapeutic agents with high reliability and specificity have been established for the treatment of TAO. The pathogenesis of TAO remains incompletely understood, highlighting the need for further investigation to develop effective therapies.

The main pathological processes of TAO are inflammatory infiltration, glycosaminoglycan accumulation, extraocular muscle thickening, adipogenesis, extensive remodeling of orbital connective tissues, and fibrosis.^8^^,^^9^ Cross-reactivity against common antigens and immune cells infiltration in the orbital is considered the initiating factor for TAO pathogenesis.^10^ In TAO, orbital fibroblasts (OFs) are considered the primary target and main effector cells,^11^^,^^12^ which overexpress a functional complex of thyroid-stimulating hormone receptor (TSHR)- insulin-like growth factor receptor (IGF-1R).^13^ When orbital fibroblasts (OFs) are activated, they secrete a range of proinflammatory and profibrotic mediators, such as interleukin (IL)-6, IL-8, and transforming growth factor (TGF)-β.^14^^,^^15^^,^^16^ Additionally, elevated levels of cytokines and growth factors, including IL-1β, IL-17A, TNF-α, IGF-1, and interferon (IFN)-γ have been detected in the orbital tissues of patients with TAO.^17^^,^^18^ These inflammatory signals are amplified in a cascade, further exacerbating inflammation and promoting hyaluronan accumulation. OFs can be divided into two subtypes based on the different expressions of CD90 (thymocyte antigen 1, Thy-1).^19^ After treatment with TGF-β, only CD90^+^ OFs were capable of differentiating into myofibroblasts, which are mainly involved in the pathogenesis of type II TAO characterized by extraocular muscle enlargement and fibrosis.^20^ When treated with peroxisome proliferator-activated receptor (PPAR)-γ agonists, CD90^−^ OFs differentiate into mature adipocytes, which are mainly involved in the pathogenesis of type I TAO associated with the expansion of the orbital adipose tissue.^19^^,^^21^ Interestingly, IGF-1 or physiological PPARγ ligands promote the differentiation of both into adipocytes when two types of cells (CD90^+/−^) are mixed in culture.^22^^,^^23^ This intricate interplay between immune signals and fibroblast fate decisions in TAO suggests that cytokines involved in both immune regulation and tissue remodeling may contribute to disease development.

IL-27, a member of the IL-12 family of cytokines, is a heterodimeric cytokine comprised of EBI3 and p28 proteins, and its receptors are composed of WSX-1 (IL-27Ra) and gp130.^24^ IL-27 is mainly produced by antigen-presenting cells (APCs), such as macrophages, monocytes, and dendritic cells,^25^ and IL-27Ra is present on diverse immune and non-immune cell types, including natural killer (NK) cells, monocytes, T and B lymphocytes, hepatocytes, tubular epithelial cells, and adipocytes.^26^^,^^27^^,^^28^ Many studies suggested that IL-27 is bifunctional in inflammatory responses (both pro-inflammatory and anti-inflammatory effects)^29^ and it is associated with a variety of autoimmune diseases, such as systemic sclerosis,^30^ ankylosing spondylitis,^31^ systemic lupus erythematosus.^32^ Beyond its role in inflammation regulation, IL-27 has demonstrated potential for anti-pulmonary fibrosis effects^33^^,^^34^ and for targeting adipocytes to influence thermogenesis and obesity.^28^

Based on our previous finding of IL-27 association with TAO,^35^ we further analyzed its expression in serum, tissues, and fibroblasts; assessed immune infiltration by bioinformatics; and investigated its regulatory effects on fibroblast activation and differentiation. Our findings suggest that IL-27 modulates autoinflammation and tissue remodeling in TAO.

## Results

### Increased serum levels of IL-27α in thyroid-associated ophthalmopathy

**Figure 1:**
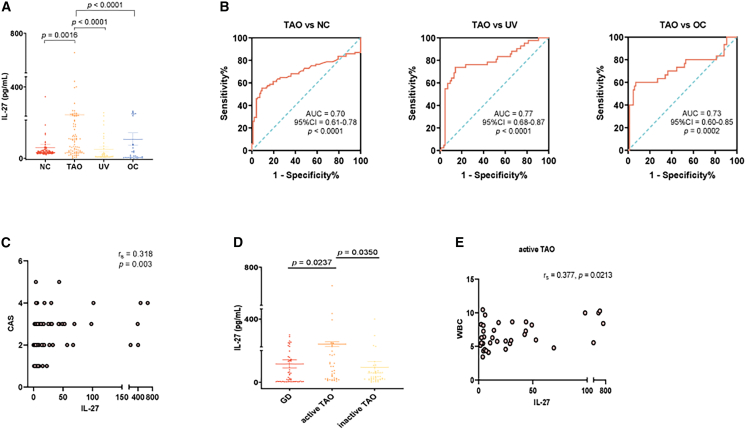
IL-27α was elevated in the serum of patients with TAO and has potential for biomarkers (A) The serum levels of IL-27α of TAO (*n* = 86) were significantly higher than NC (*n* = 97), UV (*n* = 42), and OC (*n* = 30). (B) ROC analysis of TAO and controls. (C) Correlation between IL-27 and CAS. (D) The expression of IL-27α in patients with TAO and GD (*n* = 56). (E) Correlation between IL-27α and WBC in patients with active TAO (*n* = 37). Data are presented as means ± SEM; each data point represents an individual experiment; *p* value by Kruskal-Wallis non-parametric test with Dunn’s post hoc test (A); Spearman’s correlation (C, E); Mann-Whitney test (D).

We first detected the serum expression level of IL-27α/p28 among TAO, NCs, and other autoimmune/inflammatory eye diseases (disease controls). Compared with NCs and disease controls, the serum level of IL-27α is significantly higher in patients with TAO (Figure 1A; TAO vs. NCs: *p* = 0.0016; TAO vs. UV: *p* < 0.0001; TAO vs. OC: *p* < 0.0001). In a sensitivity analysis of TAO vs. NCs, multivariable logistic regression adjusting for age and sex showed that higher IL-27 remained associated with TAO (adjusted OR per 1-unit increase, 1.026; 95% CI 1.008–1.054; *p* = 0.0267). The calibration of the age/sex-adjusted logistic model was evaluated using corrected calibration curves (Figure S1E). The baseline characteristics of the patients with TAO, NCs, and disease controls are summarized in Tables 1 and S1.

**Table 1:** The baseline characteristics of patients with TAO and NCs

Characteristics	TAO (*n* = 86)	NC (*n* = 97)	*p-*value	Reference value
Sex, m/f	29/57	42/55	0.185^a^	–
Age, y	45.94 ± 12.2	45.12 ± 14.37	0.681^b^	–
WBC, 10^9^/L	6.37 (5.65–8.25)	–	–	3.5–9.5
FT3, pg/mL	2.80 (2.445–4.670)	–	–	1.80-3.80
FT4, ng/mL	1.26 (0.96–1.955)	–	–	0.78-1.86
TSH, mIU/L	1.328 (0.068–2.911)	–	–	0.38-5.57
TgAb, IU/mL	67.18 (11.50–350.2)	–	–	0–120
TPOAb, IU/mL	50.95 (10.11–288.5)	–	–	0–35
TRAb, IU/mL	7.96 (2.34–18.03)	–	–	0–1.75
CAS, non-active/active	2.00 (2.00–3.00), 49/37	–	–	0–7
NOSPECS	4.00 (3.00–4.00)	–	–	0–6

CAS, clinical activity score; f, female; m, male; TAO, thyroid-associated ophthalmopathy; NC, normal control; y, year; FT3, free triiodothyronine, FT4, free tetraiodothyronine; TSH, thyroid-stimulating hormone; TgAb, thyroglobulin antibody; TPOAb, thyroid peroxidase autoantibody; TRAb, TSH receptor antibodies; WBC, white blood cell.

a Chi-square test.

b Student’s two-tailed *t* test.

To assess the diagnostic potential of IL-27α, receiver operating characteristic (ROC) analysis was conducted. IL-27α effectively discriminated patients with TAO from NCs with an area under the curve (AUC) of 0.70 (*p* < 0.0001). When compared with patients with other inflammatory eye diseases, IL-27 also showed moderate diagnostic performance: AUC = 0.77 for TAO vs. UV (*p* < 0.0001), and AUC = 0.73 for TAO vs. OC (*p* = 0.0002) (Figure 1B). Using the Youden index to identify data-driven thresholds, the optimal cut-off values for IL-27α were 8.484 for TAO vs. NC, 3.660 for TAO vs. UV, and 2.294 for TAO vs. OC (assay units, pg/mL). These findings suggest that IL-27 may serve as a supportive indicator to differentiate TAO from healthy individuals and, to some extent, from other orbital inflammatory conditions.

We further analyzed the relationship between IL-27α and clinical parameters of patients with TAO. The serum levels of IL-27α were positively correlated with CAS in patients with TAO and with WBC expression only in patients with active TAO (Figures 1C–1E). There was no significant correlation between other clinical parameters and IL-27α/p28 levels (Figures S1A–S1D). Due to substantial missing values in thyroid-related parameters such as TRAb, TPOAb, and TgAb, these indicators were not included in the current correlation analyses. Considering the close link between TAO and Graves’ disease (GD), we next compared serum IL-27α levels between patients with TAO and GD. IL-27α was significantly elevated in active TAO compared to patients with GD (Figure 1D), although patients with GD also exhibited increased IL-27α levels compared to NCs. These findings indicate that IL-27α alone may have limited ability to distinguish TAO from GD, particularly in subclinical or early-stage cases. Additionally, no significant difference in serum IL-27α levels was observed between steroid-treated and untreated patients with TAO (*p* = 0.186; data not shown), suggesting that recent glucocorticoid therapy did not substantially influence circulating IL-27α concentrations in this cohort.

In summary, IL-27α is upregulated in TAO, especially in active cases, and demonstrates moderate diagnostic performance in distinguishing TAO from both healthy individuals and other inflammatory eye diseases.

### Increased IL-27α/IL-27Ra expression in orbital tissues and orbital fibroblasts in thyroid-associated ophthalmopathy

**Figure 2:**
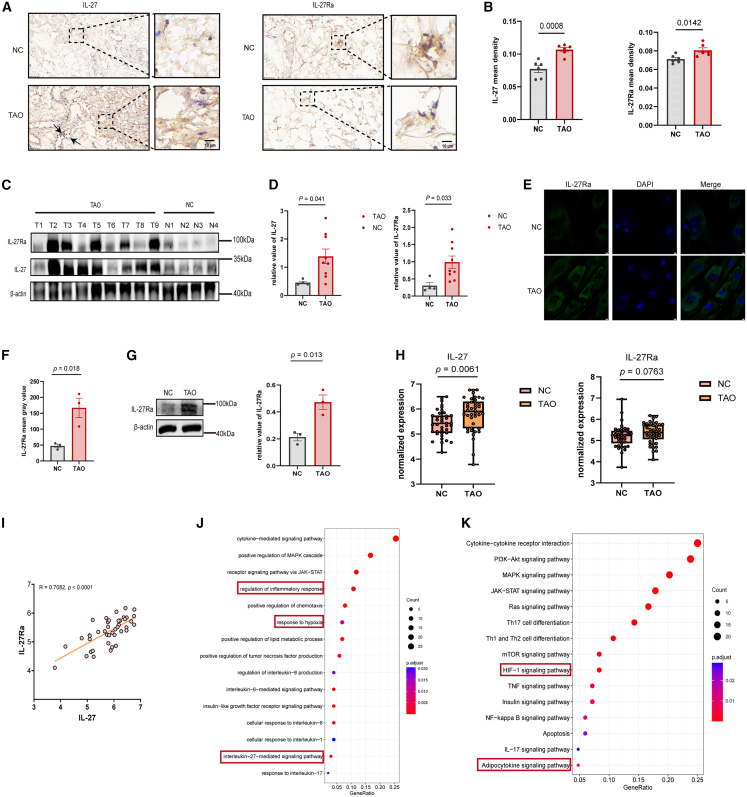
Increased IL-27α and IL-27Ra in the orbital tissues and OFs in TAO (A and B) IHC staining of IL-27α and IL-27Ra in TAO and NC orbital connective tissues (*n* = 6). Overview, 40×; insets, 200×; Arrows indicate juxtavascular clusters of nucleated cells with strong IL-27 staining, compatible with immune-cell aggregates. IL-27Ra is stronger along fibroblast-rich stromal septa; adipocytes are largely negative. Scale bars, 50 μm (40×) and 10 μm (200×). (C and D) Immunoblot analysis of the selected proteins in orbital connective tissues from TAO (*n* = 9) and NC (*n* = 4). (E and F) Immunofluorescence of IL-27Ra in OFs from TAO (*n* = 3) and NC (*n* = 3). Scale bars, 5 μm. (G) Immunoblot analysis of IL-27Ra in orbital connective tissues from TAO and NC (*n* = 3). (H and I) Transcriptome data of IL-27α and IL-27Ra in orbital tissues from the public database (TAO, *n* = 42; NC, *n* = 39); Correlation between IL-27α and IL-27Ra. (J) GO analysis of DEGs in the orbital tissues from GSE58331. (K) KEGG analysis of DEGs in the orbital tissues from GSE58331. Data are presented as means ± SEM; each data point represents an individual experiment; *p* value by unpaired two-tailed *t* test (B, D, F, G; H for IL-27); Mann-Whitney test (H for IL-27Ra); Pearson’s correlation (I).

To identify the expression of IL-27 and its receptor at the tissue level, we isolated the orbital connective tissues and cultivated primary OFs (Figures S2B and S2C) from patients with TAO and NCs. HE and MASSON staining showed significant fibrosis in patients with TAO compared with NCs (Figure S2A). We first detected the expression of IL-27α/p28 and its receptor, IL-27Ra. Immunohistochemistry showed juxtavascular and stromal (pericellular/ECM-associated) enrichment of IL-27α and stronger IL-27Ra along fibroblast-rich stromal septa in TAO than in NC, with adipocytes largely negative. Region-based mean DAB density within stromal/juxtavascular ROIs confirmed higher signals in TAO (Figures 2A and 2B). This was in line with our findings from serological studies and subsequent Western blotting (Figures 2C and 2D). We next determined the expression of IL-27Ra in OFs by immunofluorescence and Western blot, and its higher expression was observed in primary OFs from TAO (Figures 2E–2G). TAO can cause extensive pathological changes in orbital tissues, including orbital connective and lacrimal gland tissues.^36^ Therefore, we further validated the IL-27α/IL-27Ra transcript levels of TAO orbital tissues in public databases using bioinformatics methods (Figure S2D). The results showed that the expression of both IL-27α and IL-27Ra was elevated in TAO orbital tissues compared with NC, and a significant positive correlation was observed between IL-27α and IL-27Ra (Figures 2H and 2I). To ensure tissue specificity, differential gene expression and enrichment analyses were performed using only orbital connective tissue samples from the GSE58331 dataset (Figures S2D–S2G). GO/KEGG analysis revealed the enrichment of the IL-27 mediated signaling pathway and pathological changes in TAO connective tissues (Figures 2J and 2K).

Taken together, these results show increased IL-27α/IL-27Ra expression in TAO at tissue and cellular levels, involving orbital tissues and OFs.

### IL-27 suppresses the inflammation response in thyroid-associated ophthalmopathy

**Figure 3:**
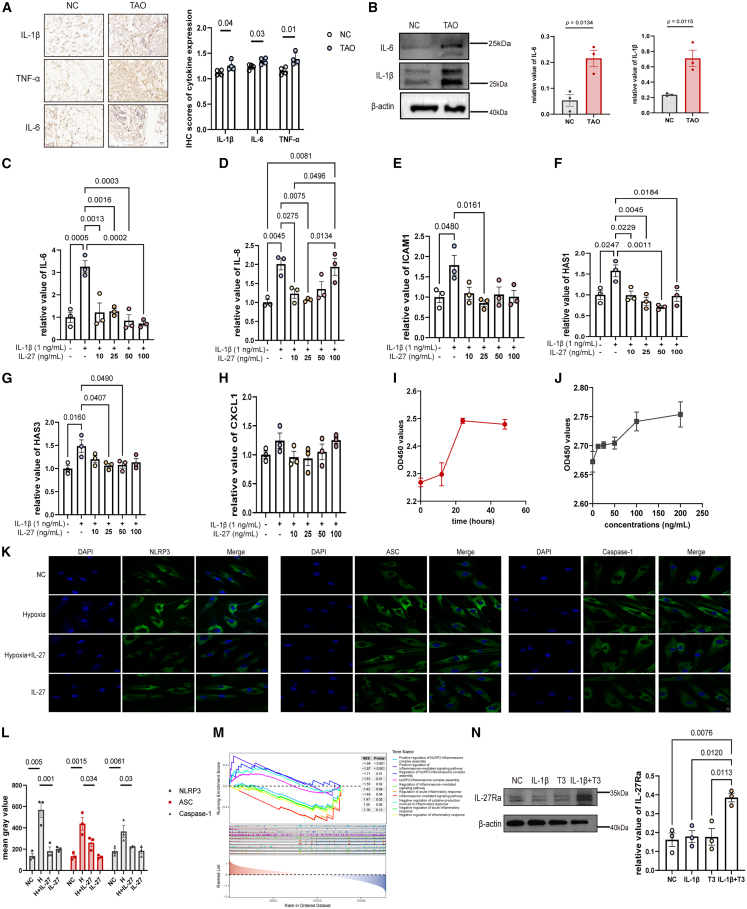
IL-27 suppressed the pro-inflammatory molecules and inflammasome in TAO (A) IHC staining of pro-inflammatory molecules in orbital connective tissues from TAO and NC and IHC score analysis (*n* = 4). Scale bars, 50 μm (40×). (B) Immunoblot analysis of the selected proteins in OFs of TAO and NC (*n* = 3). (C–H) qPCR analysis of the relative mRNA expression in TAO-OFs (*n* = 3). (I and J) CCK-8 analysis for cell viability under IL-27 treatment (*n* = 3). (K and L) Immunofluorescence for NLRP3 inflammasome in TAO-OFs (*n* = 3). Scale bars, 5 μm. (M) GSEA-GO analysis based on IL-27 expression in orbital connective tissues from GSE58331. (N) Immunoblot analysis of IL-27Ra in NC-OFs (*n* = 3). Data are presented as means ± SEM; each data point represents an individual experiment; *p* value by one way ANOVA with Tukey’s test (C–H, L, N); unpaired two-tailed *t* test (B).

IL-27 exhibits dual effects on inflammatory response in autoimmune disease.^29^ However, the corresponding effects of IL-27 in TAO remain unknown. We first detected the inflammation levels of orbital connective tissues and OFs in TAO. Higher expressions of inflammatory molecules, including IL-1β, IL-6, and TNF-α, were observed in TAO compared with NCs (Figures 3A and 3B). We next found that exogenously added IL-27 attenuated the inflammatory response induced by IL-1β on human TAO-OFs *ex vivo* at the mRNA level through inhibiting the production of pro-inflammatory molecules including IL-6, IL-8, and intercellular adhesion molecule 1 (ICAM-1), and inhibiting the production of hyaluronan synthase (HAS) (Figures 3C–3G); however, this mechanism does not seem to involve the inhibition of chemokines (Figure 3H). Administration over a time gradient (up to 48 h; 100 ng/mL) or a concentration gradient (up to 200 ng/mL) did not produce a significant inhibitory effect on cell growth (Figures 3I and 3J). NLRP3 inflammasome activation has been reported in OFs from patients with TAO.^37^ Considering the hypoxic microenvironment widely present in TAO^38^ (Figures 2J, 2K, S3A, and S3B), we next examined the effect of IL-27 on hypoxia-induced NLRP3 activation. Under hypoxic conditions, NLRP3 expression was significantly upregulated, whereas IL-27 treatment effectively attenuated this increase (Figures 3K and 3L). We further validated our findings through bioinformatics analysis based on orbital connective tissue samples, with IL-27-associated differentially expressed genes visualized in a heatmap (Figure S3C). By analysis of GSEA based on GO, we found that the decreased expression of genes associated with the positive regulation of NLRP3 inflammasome and increased expression of genes associated with the negative regulation of inflammatory response in samples with high IL-27 expression (Figure 3M), indicative of the anti-inflammatory effects of IL-27, which is consistent with our results. Moreover, in primary orbital fibroblasts from healthy controls (non-TAO), combined stimulation with IL-1β (10 ng/mL) and triiodothyronine (T3, 10 μM) for 24 h increased IL-27Ra expression as shown by Western blot (Figure 3N), whereas either stimulus alone did not produce a significant change, which suggests the IL-27 axis may require combined inflammatory and endocrine inputs.

Taken together, these results suggest that IL-27 inhibits the activation of pro-inflammatory cytokines and the NLRP3 inflammasome in TAO-OFs.

### IL-27 alleviates the fibrosis progression via the MAPK pathway in thyroid-associated ophthalmopathy

**Figure 4:**
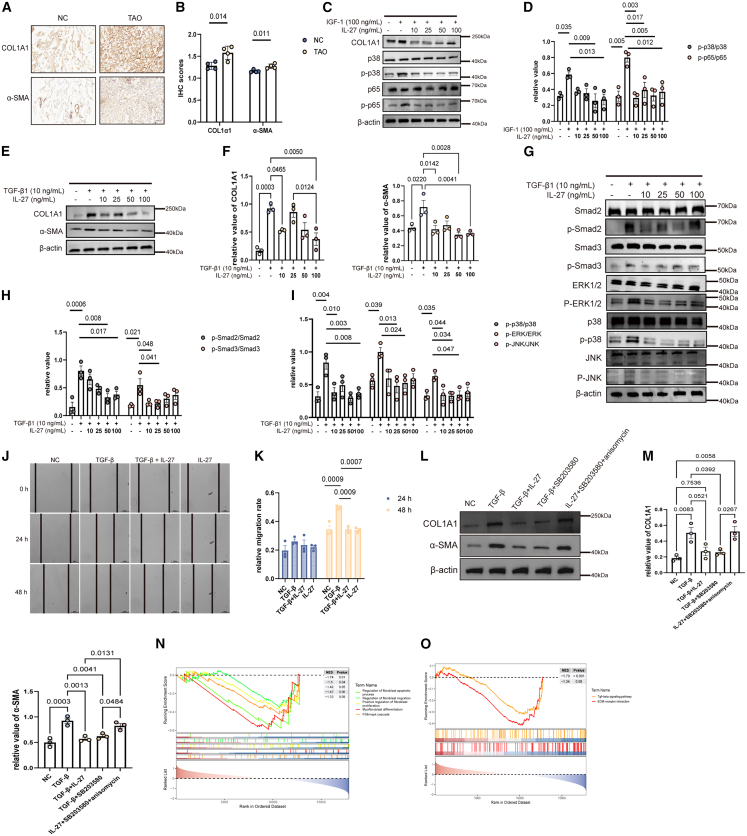
IL-27 inhibited fibrosis progression through the MAPK pathway in TAO (A and B) IHC staining for fibrosis markers (*n* = 4). Scale bars, 50 μm (40×). (C and D) Immunoblot analysis of the selected proteins under the treatment of IL-27 and IGF-1(*n* = 3). (E and F) Immunoblot analysis of the selected proteins under the treatment of IL-27 and TGF-β1 (*n* = 3). (G–I) Immunoblot analysis of the selected pathway proteins under the treatment of IL-27 and TGF-β1 (*n* = 3). (J and K) Wound-healing assay of TAO-OFs (*n* = 3). Scale bars, 500 μm. (L and M) Immunoblot analysis of the indicated proteins (*n* = 3). (N) GESA-GO analysis based on IL-27 expression in orbital connective tissues from GSE58331. (O) GESA-KEGG analysis based on IL-27 expression in orbital connective tissues from GSE58331. Data are presented as means ± SEM; each data point represents an individual experiment; *p* value by one way ANOVA with Tukey’s test (D, F, H, I, K, M); unpaired two-tailed *t* test (B).

Because fibrosis is a key pathological feature of TAO, we first assessed the canonical fibrotic markers collagen type I alpha 1 (COL1A1) and α-smooth muscle actin (α-SMA). Immunohistochemistry of orbital connective tissue (Figures 4A and 4B) showed stronger COL1A1 and α-SMA staining in TAO than in NC. Consistently, immunoblotting of primary orbital fibroblasts (Figure S4A) confirmed higher protein levels of COL1A1 and α-SMA in TAO versus NC, indicating enhanced fibroblast-to-myofibroblast transdifferentiation and fibrosis. IGF-1 and TGF-β are considered important triggering factors for extracellular matrix remodeling in TAO. We next used exogenous IGF-1 and TGF-β stimulation to simulate the fibrosis process of OFs in TAO. Upregulations of COL1A1, the phosphorylation of NF-κBp65, and p38/MAPK were noted under stimulation by IGF-1 (Figure 4C), which were consistent with a previous study.^39^ We found that IL-27 significantly inhibited collagen expression and phosphorylation of p38 and p65 (Figures 4C and 4D). Due to the exclusive expression of α-SMA in CD90^+^ OFs,^19^ we therefore isolated CD90^+^ OFs and treated them with TGF-β1 to further assess the anti-fibrotic effect of IL-27 (Figures S4B–S4E). For the stimulation of TGF-β1, increased expression of COL1A1 and α-SMA was observed, indicating the induction of fibrosis (Figures 4E and 4F). Moreover, the fibrosis related pathways TGF-β/Smad and MAPK were activated by TGF-β1 (Figures 4G–4I). A consistent downward trend in COL1A1 and α-SMA expression was observed with increasing IL-27 concentrations; however, the inhibitory effect plateaued at higher doses, suggesting a potential ceiling effect (Figures 4F and 4H–4I). Wound-healing assay was used to assess the effect of IL-27 on cell migration. TGF-β promoted the migration of TAO-OFs, and IL-27 significantly inhibited their migration rate under TGF-β treatment. No significant inhibitory effect was observed in the group of IL-27 treatment compared with NC (Figures 4J and 4K). To mechanistically link these phenotypes to proximal IL-27R signaling, we profiled acute STAT kinetics and verified IL-27Ra knockdown efficiency at both the protein and functional levels. Upon IL-27 stimulation (10 ng/mL), p-STAT1(Y701) and p-STAT3(Y705) rose rapidly with a clear peak at 5 min and declined by 15–30 min (0/5/15/30-min time course; Figures S4F and S4G). IL-27Ra knockdown efficiency was confirmed by Western blot (Figure S4J) and functionally by a significant decrease of IL-27-induced p-STAT3 at 5 min in siIL27RA + IL-27 versus siNC + IL-27 (Figure S4H). Using this 5-min peak as the canonical readout, we then performed a mechanism panel (IL-27, IL-27 + Takinib 10 μM, IL-27 + Stattic 10 μM, siIL27RA + IL-27): p-STAT3 was significantly reduced by siIL27RA and by Stattic, but unchanged by Takinib; in contrast, p-p38(Thr180/Tyr182) was significantly decreased by Takinib, while Stattic had minimal effect, and siIL27RA + IL-27 did not reach significance at this acute time point (Figure S4H). Together, these data support a parallel-branch model downstream of IL-27R/gp130 in OFs, with p38 engagement predominantly TAK1-mediated and JAK/STAT (STAT3) operating in parallel under our conditions, and we refrain from inferring a single linear hierarchy between STAT and TAK1.

To test whether p38 mediates IL-27’s anti-fibrotic effect, we used canonical modulators. Under TGF-β1, IL-27 or the p38 inhibitor SB203580 (10 μM) each reduced COL1A1 and α-SMA relative to TGF-β1 alone, whereas adding a short pulse of the p38 activator anisomycin to the TGF-β1 + IL-27 condition restored their expression (Figures 4L and 4M), supporting a p38-dependent component of the IL-27 effect. Additionally, the activation of Smad2/3 was also observed under TGF-β1 treatment and partially reduced by IL-27 (Figure 4G), suggesting that IL-27 may concurrently modulate TGF-β/Smad signaling. GSEA-GO and KEGG analysis also showed that increased expression of genes associated with the positive regulation of fibrosis was observed in samples with low IL-27 expression (Figures 4N and 4O), suggesting an antagonistic effect of IL-27 on fibrosis. Together, these findings demonstrate that IL-27 exerts anti-fibrotic effects via the MAPK pathway in TAO-OFs.

### IL-27 inhibits adipogenesis induced by multiple pathways in thyroid-associated ophthalmopathy

**Figure 5:**
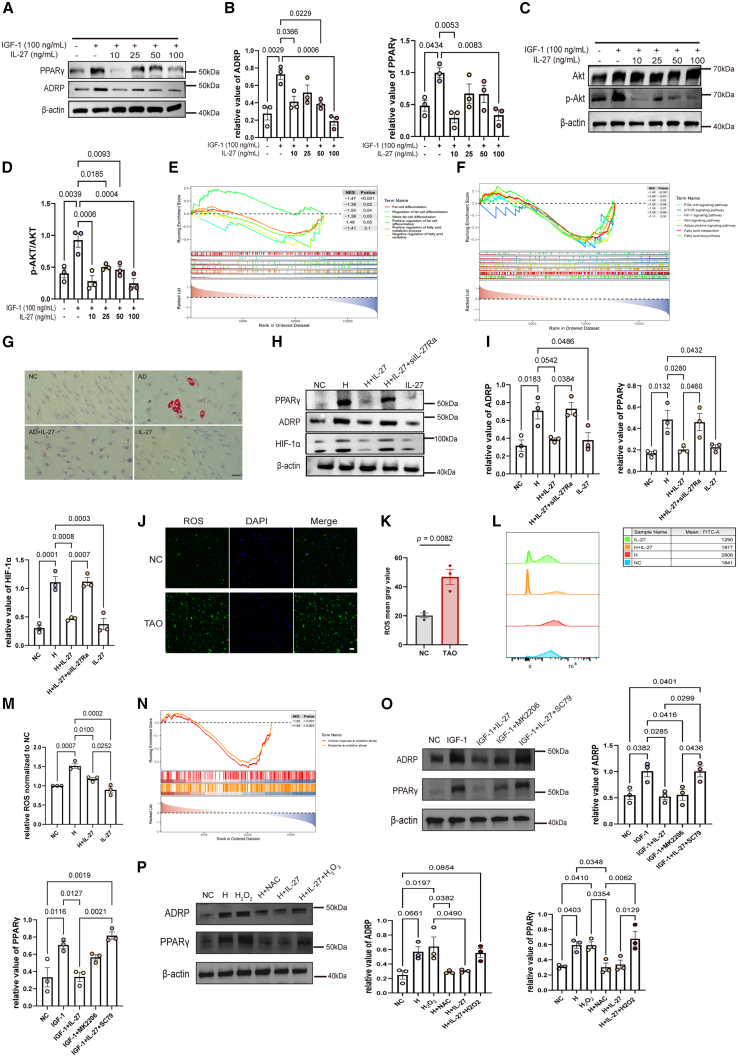
IL-27 limited adipogenesis induced by multiple pathways in TAO (A and B) Immunoblot analysis of the indicated proteins in TAO-OFs under the treatment of IGF-1 and IL-27 (*n* = 3). (C and D) Immunoblot analysis of the indicated pathway proteins (*n* = 3). (E-F) GESA-GO/KEGG analysis based on IL-27 expression in orbital connective tissues from GSE58331. (G) Oil Red O staining for TAO-OFs under adipogenic differentiation. Scale bars, 100 μm. (H and I) Immunoblot analysis of the indicated proteins (*n* = 3). (J and K) Immunofluorescence of ROS in OFs (*n* = 3). Scale bars, 50 μm. (L and M) Flow cytometry for ROS in TAO-OFs (*n* = 3). (N) GSEA-GO analysis based on IL-27 expression in orbital connective tissues from GSE58331. (O) Immunoblot analysis of the indicated pathway proteins (*n* = 3). (P) Immunoblot analysis of the indicated pathway proteins (*n* = 3). Data are presented as means ± SEM; each data point represents an individual experiment; *p* value by one way ANOVA with Tukey’s test (B, D, I, M, O, and P); unpaired two-tailed *t* test (K).

The orbital adipose tissue expansion is thought to be another important pathological feature of TAO. However, the homeostatic regulation of adipogenesis in TAO remains unknown. CD90^−^subsets of TAO-OFs are more closely related to adipogenesis,^19^ we therefore isolated CD90^−^ TAO-OFs for further study (Figure S4D). IGF-1, hypoxia, and oxidative stress contribute greatly to adipogenesis of TAO,^22^^,^^38^^,^^40^ and we explored the role of IL-27 in these processes. Four days of induction of adipose differentiation medium configured with IGF-1 instead of insulin (Methods) significantly induced the expression of adipocyte markers in CD90^−^ OFs, including PPARγ and adipose differentiation-related protein (ADRP) (Figures 5A and 5B), similar to a previous study for OFs.^22^ Of note, HIF-1α, a transcription factor implicated in promoting adipogenesis, was upregulated during IGF-1-induced adipogenic differentiation under normoxic conditions, indicating IGF-1 may induce a pseudo-hypoxic state (Figure S4I). IL-27 treatment significantly reduced adipogenic markers and HIF-1α accumulation, supporting a suppressive role for IL-27 in OF adipogenesis.

Mechanistically, IGF-1 stimulation activated both PI3K/Akt signaling, as evidenced by increased Akt phosphorylation. Exogenous IL-27 significantly blocked the phosphorylation of AKT (Figures 5C and 5D), and transcriptome-based GSEA analysis further revealed that increased gene expressions associated with the adipocytokine signaling pathway, HIF-1α pathway, and PI3K-Akt-mTOR pathway were detected in samples with low IL-27 levels, suggesting a negative regulatory effect on these pathways (Figures 5E and 5F). The anti-adipogenic effect of IL-27 was also observed under 10 days of conventional adipogenic differentiation (AD) conditions (Methods), which was detected by Oil Red O staining (Figure 5G).

A previous study demonstrated that hypoxia directly promoted adipogenesis of TAO-OFs via HIF-1α activation.^41^ To further explore the anti-adipogenic effect of IL-27 under hypoxic conditions, we treated OFs with standard adipogenic differentiation medium in the presence of hypoxia. In parallel, siRNA-mediated knockdown of IL-27Ra (Figure S4J) was performed to determine whether the observed effect was dependent on IL-27 receptor signaling. Significantly elevated expression of ADRP, PPARγ, and HIF-1α was observed during the adipogenic differentiation for 4 days (hypoxia on the fourth day), and IL-27 reduced the protein levels of these molecules (Figures 5H and 5I), indicating IL-27 inhibited adipogenic differentiation induced by hypoxia, which may be achieved by the inhibition of HIF-1α. IL-27Ra knockdown by siRNA effectively reversed this process, which indicated that IL-27Ra is necessary for IL-27 to regulate this pathological change. Oxidative stress is another important trigger for adipogenic differentiation for OFs, and hypoxia can directly induce oxidative stress.^42^ To determine whether IL-27 regulated oxidative stress, we detected ROS levels by flow cytometry and immunofluorescence. Compared with NC, significantly higher ROS was observed in the OFs of TAO under conventional cultivation (Figures 5J and 5K). The MFI of total ROS in TAO-OFs was significantly higher in hypoxia groups than in the normoxic group, and IL-27 significantly reduced the accumulation of ROS during hypoxia (Figures 5L and 5M); however, the inhibition effects were not significant under normoxic conditions. Similarly, GSEA analysis showed that samples with low IL-27 levels exhibit higher enrichment of oxidative stress-related genes than those with high IL-27 levels (Figure 5N), indicating the role of anti-oxidative stress in IL-27. To attribute pathways in adipogenesis, we used pharmacologic epistasis. In IGF-1-driven CD90^−^ OFs, IL-27 and the AKT inhibitor MK2206 each reduced ADRP and PPARγ, whereas the AKT activator SC79 restored these markers in the IGF-1 + IL-27 condition, indicating an AKT-dependent component of IL-27’s anti-adipogenic effect (Figure 5O). To test ROS causality under hypoxia, the ROS scavenger NAC phenocopied IL-27’s suppression of ADRP/PPARγ, whereas exogenous H_2_O_2_ reversed the IL-27 effect and restored these markers (Figure 5P), supporting that IL-27 suppresses hypoxia-induced adipogenesis in a ROS-dependent manner.

### Integrative transcriptomic analysis of IL-27 in orbital connective tissues in thyroid-associated ophthalmopathy

**Figure 6:**
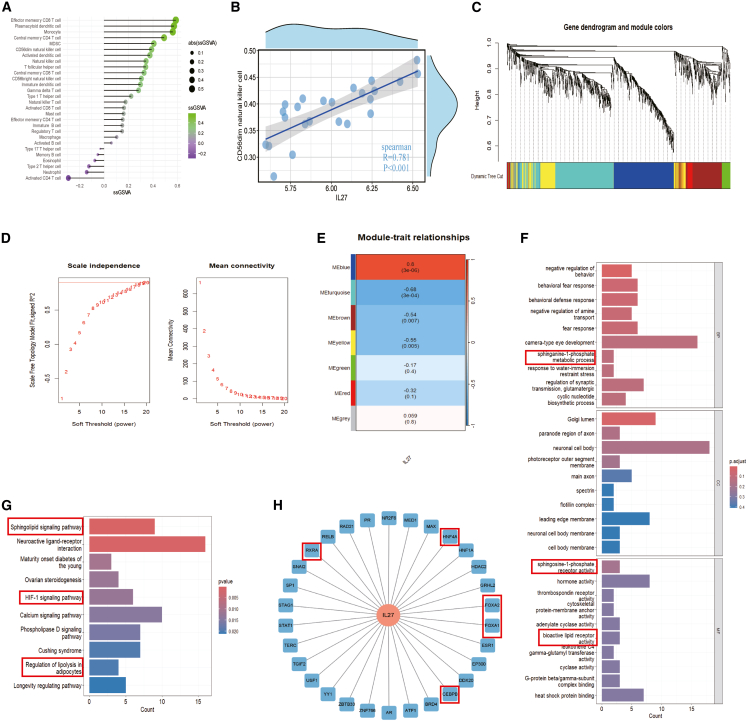
Integrative transcriptomic analysis of IL-27 in the orbital connective tissues of TAO (A and B) ssGSVA for immune infiltration; correlation between IL-27 and CD56dim natural killer cells. (C) The cluster dendrogram of WGCNA analysis. (D) The soft threshold power (left) and mean connectivity (right) of WGCNA. (E) 7 modules revealed by the WGCNA. (F and G) GO and KEGG analysis based on the blue module of WGCNA. (H) Cistrome DB analysis for transcription factor prediction.

To further investigate the function of IL-27 in the immune microenvironment of TAO orbital connective tissues. We then performed the analysis of immune infiltration and weighted gene co-expression network (WGCNA), using differentially expressed genes (DEGs) based on IL-27 expression grouping in orbital connective tissues, and the heatmap is shown in Figure S3C. Single-sample gene set variation analysis (ssGSVA) was used to assess the infiltration levels of various immune cell types, and the results revealed varying degrees of immune cell infiltration, with effector memory CD8 T cell, plasmacytoid dendritic cell, monocyte, and central memory CD4 T cell showing significantly higher enrichment scores (ECs) and positive correlation coefficients in samples with high IL-27 levels. In contrast, activated CD4 T cells, neutrophils, and type 2 T helper cells exhibited greater ECs and a negative correlation coefficient (Figure 6A). Correlation analysis revealed a significant association between IL-27 levels and CD56dim natural killer cells (Figure 6B). Previous studies have widely suggested that natural killer cells (including CD56dim natural killer cells)^43^^,^^44^ are closely related to anti-fibrosis, while plasmacytoid dendritic cells^45^ and monocytes^46^ exerted effects of anti-fibrosis in specific microenvironments. Effector memory CD8 T cells were considered associated with obesity,^47^ and natural killer cells also contributed to obesity-induced insulin resistance.^48^ Of note, type 17 T helper cells were significantly negatively correlated with the expression of IL-27, which exacerbated fibrosis in TAO.^49^

WGCNA analysis was next performed on the above DEGs. The soft-threshold power was calibrated to 8 (scale-free R2 = 0.9), and a sum of 7 modules was shown (Figures 6C–6E). Due to the significance between the bule module and IL-27, it is used for further GO/KEGG analysis. This module involved the sphingolipid signaling pathway, the HIF-1 signaling pathway, and the regulation of lipolysis in adipocytes (Figures 6F and 6G), which is closely related to adipogenesis in TAO.^50^^,^^51^ Finally, Cistrome DB analysis (correlation greater than 0.6) further predicts the regulatory relationship of the IL-27 gene (Figure 6H), including RXRA, CEBPB, FOXA1, FOXA2, and HNF4A, which are closely related to adipogenesis and fibrosis. Together, these data suggest that in addition to OFs, IL-27 may act as a cytokine secreted by immune cells to regulate the function of multiple immune cells to maintain immune homeostasis in TAO.

## Discussion

Our previous study reported the involvement of IL-12 family in the TAO, which was detected in the serum of patients.^35^ Here, we highlighted that IL-27, multi faced players in the IL-12 family, exerted a triple role of anti-inflammatory/fibrotic/adipogenic effects in TAO. Several clues drive us to conduct further research on IL-27; for example, IL-27 exhibits a dual role in several inflammatory autoimmune diseases, such as Sjogren’s syndrome, rheumatoid arthritis, inflammatory bowel disease, and systemic lupus erythematosus, where it can either promote or inhibit disease progression^52^; IL-27 attenuates pulmonary fibrosis^33^^,^^34^^,^^53^ but exacerbates skin fibrosis in systemic sclerosis^30^; IL-27 acts directly on adipocytes, enhancing thermogenesis and providing protection against diet-induced obesity^28^; IL-27 promotes lipolysis while inhibits lipogenesis, reducing lipids accumulating.^54^ These pieces of evidence suggest the powerful regulatory potential of IL-27 in autoimmune diseases.

Currently lack of sufficient understanding of the mechanisms of TAO pathogenesis limits the application of targeted biological therapy. We showed here that IL-27α/p28 and its receptor IL-27Ra (mainly IL-27) were elevated in serum and orbital connective tissues of patients with TAO, and IL-27 has the potential as a TAO biomarker. Although the ROC analysis demonstrated a statistically significant difference in serum IL-27 levels between patients with TAO and controls, the AUC (0.7–0.8) suggests only modest diagnostic accuracy, which limits the clinical utility of IL-27 as a standalone biomarker. Therefore, IL-27 may be more useful when combined with other clinical indicators or biomarkers to enhance diagnostic precision. In addition, systemic inflammatory indices (e.g., CRP) were not measured, and local or systemic inflammation could contribute to circulating IL-27, and thus TAO specificity should be interpreted with caution. IL-27Ra is primarily expressed on immune cells,^55^ and in our study, we first identified the expression of IL-27Ra on OFs, which showed higher levels in TAO than NC. Orbital inflammatory infiltration is the initial stage of TAO, mediating the activation of fibroblasts and downstream effects. At the cellular level, we observed that IL-27 reduced inflammation induced by IL-1β and formation of inflammasomes in TAO-OFs. Importantly, the anti-inflammatory effect of IL-27 did not show a linear dose-response relationship, with the highest concentration exhibiting a reduced suppressive effect on cytokine expression. This may reflect receptor saturation, feedback inhibition, or biphasic signaling dynamics commonly observed in regulatory cytokines. Immune infiltration analysis also revealed the negative regulation of neutrophils by IL-27. Notably, IL-27 has dual effects of anti-inflammation and pro-inflammation,^52^ and our results showed IL-27 exerted the anti-inflammatory role in TAO. Interestingly, the expression of IL-27 in the same disease exhibits ethnic, racial, and regional specificity,^52^ we reasoned that the anti-inflammatory or pro-inflammatory properties of IL-27 may exhibit positive or negative feedback characteristics in different immune microenvironments.

IL-27 is primarily produced by antigen-presenting cells such as monocytes, macrophages, and dendritic cells. In APCs, IL-27 (p28+EBI3) is induced by TLR ligands, CD40 ligation, and pro-inflammatory cytokines (IFN-γ, type I IFN, TNF-α, and IL-1β). p28 is regulated by IRF1 and NF-κB and is further enhanced through the IFN-γ/JAK-STAT1 pathway acting upstream of IRF1, whereas EBI3 is predominantly NF-κB-responsive.^56^ In TAO, systemic immune activation and local infiltration of these immune cells likely contribute to elevated serum IL-27 levels, particularly in active disease. While IL-27 exhibits anti-inflammatory and antifibrotic effects on orbital fibroblasts *in vitro*, its paradoxical increase in patients may reflect an endogenous compensatory response aimed at limiting excessive immune activation-similar to observations in systemic lupus erythematosus and rheumatoid arthritis.^57^^,^^58^ Although IL-27 levels are elevated systemically in patients with TAO, exogenous IL-27 was still required to elicit anti-inflammatory and antifibrotic effects *in vitro*. This may reflect limited local bioavailability or impaired responsiveness to IL-27 within the orbital microenvironment. Our serum assay measures IL-27α/p28, whereas all mechanistic experiments use heterodimeric IL-27 (p28+EBI3) via IL-27R. Because p28 can act differently from the heterodimer in a context-dependent manner, we interpret the serum finding as p28-related and anchor causality to the heterodimer data in OFs. Practically, serum p28 may not linearly reflect heterodimer activity in orbit. Future work will co-measure EBI3 or use heterodimer-specific assays and test recombinant p28 directly in OFs.

Fibrosis and adipogenesis of orbital tissues are hallmarks of TAO, and CD90-defined heterogeneity guides OF fate.^19^ In CD90^+^ TAO-OFs, TGF-β1 increased COL1A1 and α-SMA and promoted migration, whereas IL-27 reduced these readouts. Mechanistically, SB203580 phenocopied the anti-fibrotic effect of IL-27, and a 30-min anisomycin pulse restored COL1A1/α-SMA in the TGF-β1 + IL-27 condition, supporting a p38-dependent component of IL-27’s anti-fibrotic action. In CD90^−^ TAO-OFs, IGF-1 upregulated ADRP and PPARγ; both IL-27 and the AKT inhibitor MK2206 reduced these markers, while the AKT activator SC79 rescued them, indicating the AKT-dependent suppression of IGF-1-driven adipogenesis. Given that hypoxia and oxidative stress are major drivers of adipogenic differentiation in TAO-OFs,^40^ we evaluated the effect of IL-27 under hypoxia: IL-27 reduced ADRP and PPARγ; NAC produced a similar decrease, whereas H_2_O_2_ restored these markers in IL-27-treated cells, indicating that IL-27’s suppression of hypoxia-induced adipogenesis is ROS-dependent. Notably, WGCNA analysis showed that the expression of IL-27 is highly correlated with sphingosine-1-phosphate, which is shown to have a dual effect of regulating fibrosis and adipogenesis in TAO.^50^^,^^51^ This may be a key research direction for the future. Collectively, p38 activity in OFs is set by TAK1 input and stress cues—TAK1 inhibition lowers p-p38 and SB203580 blocks p38 output, while IL-27 alone does not raise p-p38 acutely. In parallel, STAT3 likely induces SOCS-type brakes that dampen profibrotic cytokine/growth-factor signaling and DUSP phosphatases that dephosphorylate MAPKs, including p38, thereby aligning with the observed reduction in COL1A1 and α-SMA. IL-27-associated lowering of ROS would further lessen stress-activated p38/MAPK tone and its crosstalk with TGF-β, reinforcing the attenuation of fibrosis markers in CD90^+^ OFs. In the adipogenic setting driven by IGF-1 in CD90^−^ OFs, STAT3 engagement likely modulates the IGF-AKT axis, while reduced ROS under hypoxia diminishes pro-adipogenic stress inputs. Together, these mechanisms converge on decreased matrix output in CD90^+^ cells and restrained adipogenesis in CD90^−^ cells without requiring a single STAT-p38 hierarchy.

T cell-mediated immunity is strongly implicated in the progression of TAO.^59^The imbalance of the Th1/Th2 or Th17/Treg ratio determines the expansion of inflammation and dynamics of fibrosis and adipogenesis.^60^ IL-27 plays a crucial role in modulating the immune microenvironment. Previous studies have shown that IL-27 negatively regulates the development of Th17 cells during central nervous system inflammation^61^ and dendritic cell-derived IL-27 p28 modulates T cell responses in acute graft-versus-host disease.^62^ We indicated that there is a close relationship between IL-27 levels and enrichment of different types of T cells in TAO. The study of the regulation of IL-27 on T cell homeostasis in TAO may uncover greater biotherapy potential.

Targeted biological therapy is receiving increasing attention in TAO therapy. Previous research mainly focuses on a single direction of either fibrosis or adipogenesis, and we identified a target with multi-directional regulatory function and restricted disease progression. In conclusion, these experimental results support the idea that IL-27, as a powerful balancer, could be instrumental in the progression of TAO. These findings may inform the development of novel biologic therapies for TAO.

### Limitations of the study

We acknowledge some limitations of the current study. First, our research is mainly based on the biological effects of IL-27 in TAO; however, CD34^+^ OFs, subgroups derived from peripheral blood in the orbit of TAO, should be further investigated. Another limitation of this study is the absence of *in vivo* validation. Although *in vitro* assays and transcriptomic analysis provided mechanistic clues, these findings need to be confirmed in animal models.

## Resource availability

### Lead contact

Further information and requests for resources should be directed to and will be fulfilled by the lead contact, Longqian Liu (b.q15651@hotmail.com).

### Materials availability

• This study did not generate new unique reagents.
• Primary human orbital fibroblasts were obtained under institutional approval and informed consent; due to consent and privacy restrictions, these primary cells are not available for distribution.
• All siRNAs used in this study were purchased from commercial vendors; sequences and catalog numbers are listed in the key resources table.

### Data and code availability

• The transcriptome data used in this study were obtained from the publicly accessible database GEO. Further source data supporting the findings of this study are available from the lead contact upon reasonable request.
• This article does not report any original code. All analyses were performed using standard software and R packages as detailed in the STAR Methods section.
• Any additional information required to reanalyze or use the data reported in this article is available from the lead contact upon request.

## Acknowledgments

This work was supported by the 10.13039/501100001809National Natural Science Foundation of China (grant number 82070996) and the Fang Qianxun-Tang Zeyuan Ophthalmic Clinical Medicine Charity Project (0040206107039).

The authors would like to thank Jinkui Pi, Shuxia Zhang, Jiayi Xu, Jinhan Zhou, and Mingjie Xu from the Core Facilities of West China Hospital, Sichuan University, for their technical assistance with cell-based assays. We also gratefully acknowledge the support of the Department of Ophthalmology and the Public Experimental Platform of the Affiliated Hospital of Southwest Medical University.

## Author contributions

P. Zhang performed, analyzed the experiments, and wrote the article; X. Wang participated in the experiment; N. Lu and Y. Nie were involved in the conceptualization of the article; X. Zhang and L. Liu supervised the project and provided ideas.

## Declaration of interests

The authors declare no conflict of interest.

## STAR★Methods

### Key resources table

**Table:** 

REAGENT or RESOURCE	SOURCE	IDENTIFIER
**Antibodies**		

IL-27 Monoclonal antibody	Proteintech	Cat# 66164-1-Ig
Collagen Type I Mouse Monoclonal antibody	Proteintech	Cat# 67288-1-Ig
NF-κBp65 Polyclonal antibody	Proteintech	Cat# 10745-1-AP
ADPR Recombinant antibody	Proteintech	Cat# 80362-2-RR
Smooth muscle actin specific Recombinant antibody	Proteintech	Cat# 80008-1-RR
PPAR Gamma polyclonal antibody	Proteintech	Cat# 16643-1-AP
TNF-alpha Polyclonal antibody	Proteintech	Cat# 17590-1-AP
IL-1 beta Polyclonal antibody	Proteintech	Cat# 16806-1-AP
IL-6 Polyclonal antibody	Proteintech	Cat# 21865-1-AP
Multi-rAb HRP-Goat Anti-Rabbit Recombinant Secondary Antibody (H + L)	Proteintech	Cat# RGAR001
Multi-rAb HRP-Goat Anti-Mouse Recombinant Secondary Antibody (H + L)	Proteintech	Cat# RGAM001
Multi-rAb™ CoraLite® Plus 488-Goat Anti-Rabbit Recombinant Secondary Antibody (H + L)	Proteintech	Cat# RGAR002
GAPDH Monoclonal antibody	Proteintech	Cat# 60004-1-Ig
STAT3 Polyclonal antibody	Proteintech	Cat# 10253-2-AP
Anti-WSX-1 antibody	Abcam	Cat# ab281998
β-Actin Rabbit mAB	ABclonal	Cat# AC026
Phospho-NF-κB p65 Rabbit mAb	Cell Signaling Technology	Cat# 3033
Smad2 (D43B4) XP® Rabbit mAb	Cell Signaling Technology	Cat# 5339
Phospho-SMAD2 (Ser465/467) (138D4) Rabbit mAb	Cell Signaling Technology	Cat# 3108
SMAD3 (C67H9) Rabbit mAb	Cell Signaling Technology	Cat# 9523
Phospho-SMAD3 (Ser423/425) (C25A9) Rabbit mAb	Cell Signaling Technology	Cat# 9520
Akt (pan) (C67E7) Rabbit mAb	Cell Signaling Technology	Cat# 4691
Phospho-Akt (Ser473) (D9E) XP® Rabbit mAb	Cell Signaling Technology	Cat# 4060
Phospho-Stat3 (Tyr705) (D3A7) XP® Rabbit mAb	Cell Signaling Technology	Cat# 9145
P38 MAPK Ab	Affinity Biosciences	Cat# AF6456
Phospho-p38 MAPK (Thr180/Tyr182) Ab	Affinity Biosciences	Cat# AF4001
ERK1/2 Ab	Affinity Biosciences	Cat# AF0155
Phospho-ERK1/2 (Thr202/Tyr204) Ab	Affinity Biosciences	Cat# AF1015
JNK1/2/3 Ab	Affinity Biosciences	Cat# AF6319
Phospho-JNK1/2/3 (Thr183+Tyr185) Ab	Affinity Biosciences	Cat# AF3318
HIF-1α Polyclonal Antibody	Immunoway	Cat# YT2133
FITC Mouse Anti-Human CD90	BD Bioscience	Cat# 555595
IL27R Rabbit pAb	Bioss	Cat# bs-2711R
Phospho-STAT1 (S727) Recombinant Rabbit Monoclonal Antibody	HUABIO	Cat# ET1611-20
KD-Validated Anti-STAT1 Recombinant Rabbit Monoclonal Antibody	GenuIN	Cat# 61713

**Biological samples**		

Human peripheral blood serum (TAO, GD, UV, OC, NC)	Affiliated Hospital of Southwest Medical University; This study	IRB: KY2021265; KY2022287
Human orbital adipose/connective tissue	Affiliated Hospital of Southwest Medical University; This study	IRB: KY2021265; KY2022287

**Chemicals, peptides, and recombinant proteins**		

Insulin	TargetMol	Cat# T8221
HumanKine® recombinant human TGF beta 1 protein	Proteintech	Cat# HZ-1011
Recombinant Human LR3 Insulin-like Growth factor-1	PrimeGene	Cat# 105-03
HumanKine® recombinant human IL-27 protein	Proteintech	Cat# HZ-1275
Recombinant Human IL-1 beta	novoprotein	Cat# GMP-CG93
Dulbecco’s Modified Eagle Medium	Gibco	Cat# C11885500BT
TRIzol reagent	Thermo	Cat# 15596018CN
Pantothenic acid	Sigma	Cat# P5155
Biotin	Sigma	Cat# B4639
Triiodothyronine	Sigma	Cat# T2877
Transferrin	Sigma	Cat# T8158
IBMX	Sigma	Cat# I5879
Dexamethasone	Sigma	Cat# D4902
Carbaprostacyclin	Cayman	Cat# 18210
Fetal bovine serum	TransGen	Cat# FS201-02
DAPI	Beyotime	Cat# C1006
MK2206	TargetMol	Cat# T62609
SC79	TargetMol	Cat# T2274
N-acetyl-L-cysteine	Solarbio	Cat# IA0050
Hydrogen peroxide	Aladdin	Cat# H433857
SB203580	TargetMol	Cat# T1764
Anisomycin	TargetMol	Cat# T6758
Takinib	TargetMol	Cat# T4264
Stattic	TargetMol	Cat# T6308

**Critical commercial assays**		

ROS Assay Kit (FITC)	Beyotime	Cat# S0033S-1
cDNA Synthesis Kit	TransGen	Cat# AT311
TransScript® RT SuperMix Kit	TransGen	Cat# AU341
ELISA kit	Cusabio	Cat# CSB-E08464h
Cell Counting Kit-8	MedChemExpress	Cat# HY-K0301

**Deposited data**		

GSE58331; GSE105149; GSE185952 (public datasets analyzed)	NCBI GEO	GSE58331; GSE105149; GSE185952

**Experimental models: Cell lines**		

Primary human orbital fibroblasts	Derived from donor orbital tissue in this study	IRB: KY2021265; KY2022287

**Oligonucleotides**		

siRNA targeting sequence:IL-27Ra (5′-UGGAGAAAGAAGAGGAUUU-3′	Sango Biotech	N/A
siRNA negative control (5′-UUCUCCGAACGUGUCACGU-3′)	Sango Biotech	N/A
IL-6 F: TTCGGTCCAGTTGCCTTCTCC; R: TCTGAAGAGGTGAGTGGCTGTC	Sango Biotech	N/A
IL-8 F: CTCTTGGCAGCCTTCCTGATTTC; R: GGGTGGAAAGGTTTGGAGTATGTC	Sango Biotech	N/A
CXCL1 F: GCTGCTCCTGCTCCTGGTAG; R: CGTTCACACTTTGGATGTTCTTGG	Sango Biotech	N/A
ICAM1 F: ACCTATGGCAACGAC TCCTTCTC; R: GTGTCTCCTGGC TCTGGTTCC	Sango Biotech	N/A
HAS1 F: GCTGACCATCTCCGCCTACC; R: GTTGCCATCCACCACCATGAG	Sango Biotech	N/A
HAS3 F: AGCGTGCGGTACTGGATGG; R: ACTGCTGGAGGAGGCTGTTG	Sango Biotech	N/A
GAPDH F: CAGGAGGCATTGCTGATGAT; R: GAAGGCTGGGGCTCATTT	Sango Biotech	N/A

**Software and algorithms**		

GraphPad Prism software	version 10.1.2	https://www.graphpad.com/
IBM SPSS Statistics	version 29.0.1.0	https://www.ibm.com/products/spss-statistics
R	version 4.4.0	https://www.r-project.org/
ImageJ	Wayne Rasband	https://imagej.net

### Experimental model and study participant details

#### Human participants

Participants were recruited at the Departments of Endocrinology, Ophthalmology, and Physical Examination, Affiliated Hospital of Southwest Medical University. Serum cohorts included TAO (*n* = 86), normal controls (NC; *n* = 97), uveitis (UV; *n* = 42), orbital cellulitis (OC; *n* = 30), and Graves’ disease without TAO (GD; *n* = 56). A subset of serum IL-27 data (TAO *n* = 75; NC *n* = 90; GD *n* = 55; UV *n* = 28) was reported previously; we extended the dataset with 11 TAO, 7 NC, 1 GD, 4 UV, and 30 OC additional cases. TAO activity was defined using EUGOGO Clinical Activity Score (CAS): active (CAS ≥3) vs. inactive (CAS <3). Inclusion/exclusion criteria are detailed in the Results/Methods; briefly, exclusions included pregnancy/lactation, other autoimmune/chronic inflammatory diseases, recent major trauma (≤3 months), and active infection (within 2 weeks). Clinical assessments followed routine practice; orbital MRI was not uniformly available. Demographics for serum cohorts are summarized in Table 1.

#### Human tissue donors

Orbital adipose/connective tissue was obtained during decompression surgery from TAO patients (*n* = 10; 8 active, 2 inactive) and from cosmetic procedures in NC donors (*n* = 8). Donors were euthyroid, stable on antithyroid therapy, and free of immunosuppressants, corticosteroids, or radiotherapy for ≥3 months. Each specimen was divided for snap-freezing, fixation, and primary culture. Demographics and clinical characteristics for tissue donors are provided in Table 2.

**Table 2:** The baseline characteristics of orbital tissue donors

Characteristics	TAO (*n* = 10)	NC (*n* = 8)	*p*-value
Sex, m/f	3/7	4/4	0.6305^a^
Age	47.70 ± 10.89	45.63 ± 21.85	0.7955^b^
WBC, 10^9^/L	6.683 ± 2.107	–	–
FT3, pg/mL	2.255 (1.975–2.808)	–	–
FT4, ng/mL	1.050 (0.733–1.450)	–	–
TSH, mIU/L	2.792 (1.507–5.249)	–	–
Smoking	3/10	3/8	–
Previous treatment	GC, 8/10	–	–
Surgical treatment	decompression	blepharoplasty	–
CAS, non-active/active	3.50 (2.75–5.00), 2/8	–	–
NOSPECS	4.50 (3.75–6.00)	–	–

CAS, clinical activity score; f, female; m, male; TAO, thyroid-associated ophthalmopathy; NC, normal control; y, year; FT3, free triiodothyronine, FT4, free tetraiodothyronine; TSH, thyroid-stimulating hormone; TgAb, thyroglobulin antibody; TPOAb, thyroid peroxidase autoantibody; TRAb, TSH receptor antibodies; WBC, white blood cell.

a Fisher exact test.

b Student’s two-tailed *t* test.

#### Primary cell cultures

Primary orbital fibroblasts (OFs) were established from minced orbital tissue and cultured in DMEM (Gibco) with 10% FBS (Gibco) at 37 °C, 5% CO_2_. Medium was refreshed every 2–3 days following cell outgrowth. OFs were used at passages 3–8. Donor sex for primary cells was not consistently recorded (see “sex and gender analysis” and limitations of the study). All primary cultures were routinely screened and tested negative for mycoplasma contamination (method: PCR-based assay or MycoAlert; specify the actual kit used). Cell line authentication is not applicable because only primary cultures were used.

#### Cell lines

Not applicable.

#### Animals

Not applicable.

#### Plants/Microbe strains

Not applicable.

#### Sex and gender analysis

For serum cohorts, associations were modeled with multivariable logistic regression adjusted for age and sex (see results). Gender identity was not collected.

#### Ethics approval and consent

Procedures conformed to the Declaration of Helsinki and were approved by the Medical Ethics Committee of Southwest Medical University (KY2021265; KY2022287). Written informed consent was obtained from all participants.

### Method details

#### Cytokine and growth factor treatments

OFs were stimulated with recombinant human (rh) TGF-β1 (10 ng/mL; Proteintech), IGF-1 (100 ng/mL; PrimeGene), IL-1β (1, 10 ng/mL; Novoprotein), and IL-27 (0, 10, 25, 50, 100 ng/mL; Proteintech). Cells were maintained in DMEM ±10% FBS as indicated.

#### Small-molecule modulators

Recommended conditions (general). MK2206 (AKT inhibitor), SC79 (AKT activator), N-acetyl-L-cysteine (NAC), hydrogen peroxide (H_2_O2), SB203580 (p38 inhibitor), anisomycin (p38/JNK activator), takinib (TAK1 inhibitor), and Stattic (STAT3 inhibitor) were used as follows: MK2206, 1 μM, 60 min pre-treatment, maintained; SC79, 10 μM, 30 min pre-treatment, maintained; NAC, 5 mM, 60 min pre-treatment, maintained; H_2_O_2_, 10 μM; SB203580, 10 μM, 60 min pre-treatment, maintained; Anisomycin (20 ng/mL) was applied at t = 4 h for 30 min, then washed once and medium replaced. Unless otherwise noted, compounds were added at the indicated pre-treatment times and cells were then stimulated with IL-27 or other cytokines/growth factors (e.g., TGF-β1, IGF-1, insulin); cultures proceeded to a total exposure of 24 h for all agents except H_2_O_2_ and HAC, which was maintained for 48 h. Acute IL-27 signaling (5-min) assays (p-STATs and p-p38). For short-kinetics readouts, cells were pre-treated for 60 min with Stattic (10 μM) and/or takinib (10 μM), followed by IL-27 stimulation for 5 min (at the indicated concentration).

Preparation. Stocks of Stattic, MK2206, SC79, SB203580, and anisomycin were prepared in DMSO and stored at −20 °C protected from light; NAC was prepared fresh in sterile water (pH adjusted as needed); H_2_O_2_ working solutions were prepared fresh in culture medium immediately before use. The final DMSO concentration was ≤0.1% (v/v).

#### Adipogenic differentiation protocols

Serum-free DMEM-based media contained pantothenic acid (17 μM), biotin (33 μM), triiodothyronine (0.2 nM), transferrin (10 μg/mL), IBMX (0.1 mM), dexamethasone (1 μM) (all Sigma), and carbaprostacyclin (cPGI_2_; 0.2 μM; Cayman). Two induction systems were used: IGF-1 (10 ng/mL) or insulin (1 μM; TargetMol). IL-27 was added to test context dependence. IBMX and dexamethasone were included only during the first 4 days; media were changed every 3 days for 4–10 days.

#### Serum IL-27 quantification (ELISA)

Serum IL-27 was measured using a commercial ELISA kit (Cusabio) according to the manufacturer’s instructions; absorbance at 450 nm was read on a microplate reader.

#### RNA isolation and qRT-PCR

Total RNA from OFs was extracted with TRIzol (Thermo). cDNA was synthesized (TransGen cDNA Synthesis Kit) and qRT-PCR performed with TransScript RT SuperMix (TransGen) on an ABI 7500 (Thermo). ACTB was the reference gene. Relative expression was calculated by 2ˆ−ΔΔCt. Primer sequences are listed in key resources table.

#### Western blotting

Cells were lysed in RIPA buffer (Cell Signaling Technology); protein concentration was determined by Bradford assay (Bio-Rad). Equal protein was resolved by SDS-PAGE and transferred to PVDF (Millipore). Membranes were blocked (5% skim milk, 1 h, RT), incubated with primary antibodies (overnight, 4 °C; see key resources table), washed with TBST, and incubated with HRP-conjugated secondaries (1 h, RT). Signals were captured on a chemiluminescence imager (Invitrogen) using OriScience reagents and quantified in ImageJ (NIH).

#### Immunohistochemistry

Paraffin sections were dewaxed, rehydrated, subjected to high-pH antigen retrieval, and blocked in 3% BSA. Primary antibodies: IL-27 (Proteintech), IL-27Ra (Bioss), IL-6 (Proteintech), IL-1β (Proteintech), HIF-1α (Immunoway), TNF-α (Proteintech) (overnight, 4 °C). Sections were washed, incubated with secondary antibody (Cell Signaling Technology), and developed with DAB.

#### Immunofluorescence

OFs (∼1×10^5^ cells/well, 6-well plates) were fixed in 4% paraformaldehyde (30 min, RT), permeabilized with 0.1% Triton X-100, and blocked with 1% BSA. Primary antibody: IL-27Ra (Bioss) (overnight, 4 °C). Alexa Fluor 488-conjugated secondary antibody (Cell Signaling Technology) was used as secondary. Nuclei were counterstained with DAPI (Beyotime, 10 min). Images were acquired on an Olympus fluorescence microscope.

#### Flow cytometry

OFs were resuspended in stain buffer (BD) and incubated with FITC-*anti*-human CD90 (Thy-1; BD) (15 min, 4 °C). Intracellular ROS was detected with DCFH-DA (Beyotime) (30 min, 37 °C, dark). Acquisition/sorting used a BD FACSMelody. Mean fluorescence intensity (MFI) was analyzed in FlowJo (TreeStar). Fluorochromes are listed in key resources table.

#### Cell viability assay (CCK-8)

OFs (4×10^3^ cells/well, 96-well plates) were treated with rhIL-27 (0, 12.5, 25, 50, 100 ng/mL) for 48 h, or with 100 ng/mL for 0, 12, 24, 48 h. CCK-8 reagent was incubated for 4 h at 37 °C and OD_450_ measured (Thermo microplate reader).

#### Wound-healing migration assay

Confluent OF monolayers were scratched with a sterile 200 μL tip, washed with PBS, and incubated in DMEM +1% FBS with or without TGF-β (10 ng/mL) and rhIL-27 (100 ng/mL). Images were captured at 0, 24, and 48 h; migration distance was quantified in ImageJ.

#### Oil Red O staining

Adipocyte-induced OFs were fixed (4% formaldehyde, 15 min, RT) and stained with Oil Red O (30 min). Stained cells were imaged on an Olympus microscope.

#### siRNA transfection

siRNAs targeting IL-27Ra (5′-UGGAGAAAGAAGAGGAUUU-3′) and negative control (5′-UUCUCCGAACGUGUCACGU-3′) (Sango Biotech) were transfected into OFs (5×10^5^ cells/well; ∼70% confluence; 6-well plates) with Lipofectamine 3000 (Invitrogen) per the manufacturer’s protocol.

### Bioinformatic analyses

Public microarray datasets were retrieved from GEO (GSE58331, GSE105149, GSE185952). Processing used R 4.4.0. Normalization used standard pipelines; batch correction used ComBat (“sva”). Differential expression used “limma” with thresholds: orbital connective + lacrimal gland |log_2_FC|>0.5, *p* < 0.05; orbital connective only |log_2_FC|>1, *p* < 0.05. Heatmaps and volcano plots used “pheatmap” and “ggplot2”. Immune-related genes were from ImmPort; intersections with DEGs defined immune-related DEGs via “ggvenn”. GO, KEGG, and GSEA used “clusterProfiler”. Immune infiltration used ssGSVA; WGCNA identified gene modules. Workflow is shown in Figure S2.

### Quantification and statistical analysis

All analyses used GraphPad Prism 10.1.2, SPSS 29.0.1.0, ImageJ 1.54days and R 4.4.0. Data normality was assessed by Shapiro-Wilk.

**Multiple groups:** one-way ANOVA with Tukey’s post hoc (normal) or Kruskal–Wallis with Dunn’s post hoc (non-normal).

**Two groups:** unpaired Student’s *t* test (normal) or Mann–Whitney U (non-normal).

**Categorical data:** Chi-Square Test.

**Correlations:** Pearson (normal) or Spearman (non-normal).

Two-sided *p* < 0.05 was considered significant. Data are presented as the mean ± the standard error of the mean (SEM), unless otherwise noted.
