# Supplementary material for: Interleukin-27 is a multitarget regulator of fibroblast remodeling in thyroid-associated ophthalmopathy
Source: iScience. 2025 Nov 10;28(12):113982. doi: 10.1016/j.isci.2025.113982 (PMC12682014; doi:10.1016/j.isci.2025.113982)
Supplement: Document S1. Figures S1–S4 and Table S1 [file mmc1.pdf]

iScience, Volume *28*

Supplemental information

Interleukin-27 is a multitarget regulator of fibroblast remodeling in thyroid-associated ophthalmopathy

Pengbo Zhang, Xiaofang Wang, Nanji Lu, Yan Nie, Xibo Zhang, and Longqian Liu

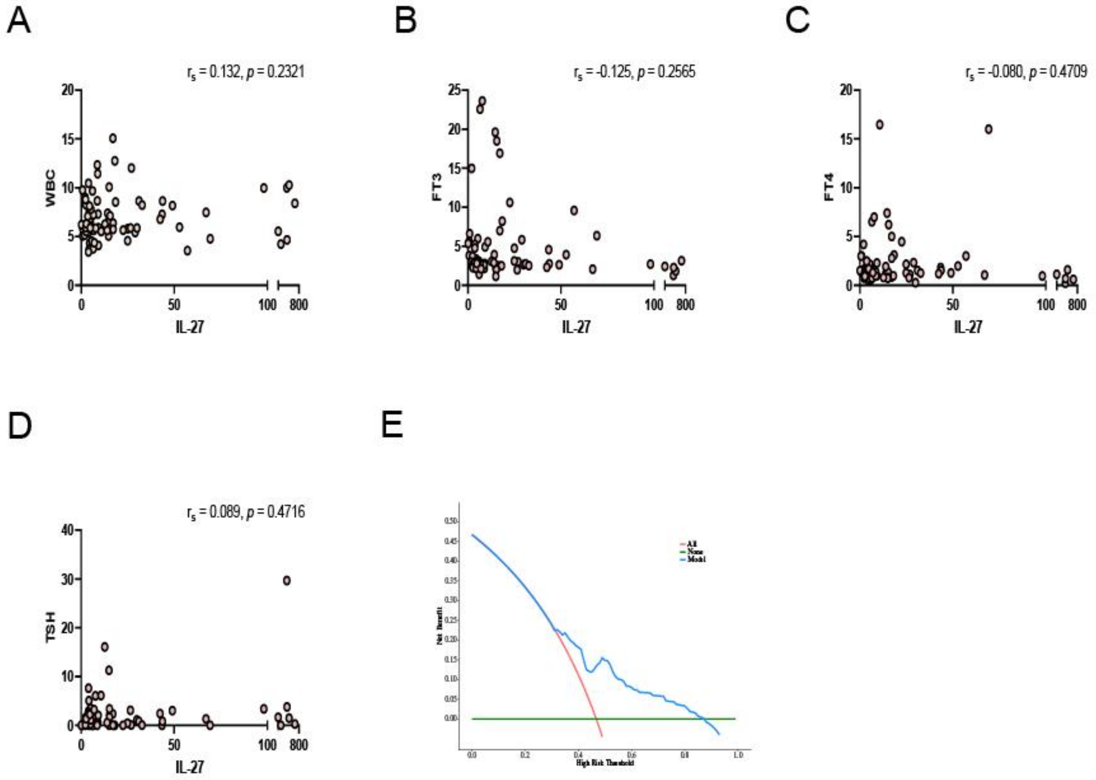

Fig.S1 Correlations between IL-27 and other clinical parameters in TAO. (A) The correlation between IL-27 and WBC. (B) The correlation between IL-27 and FT3. (C) The correlation between IL-27 and FT4. (D) The correlation between IL-27 and TSH. (E) corrected calibration curves for the age/sex-adjusted logistic model. Spearman's correlation (A-D).

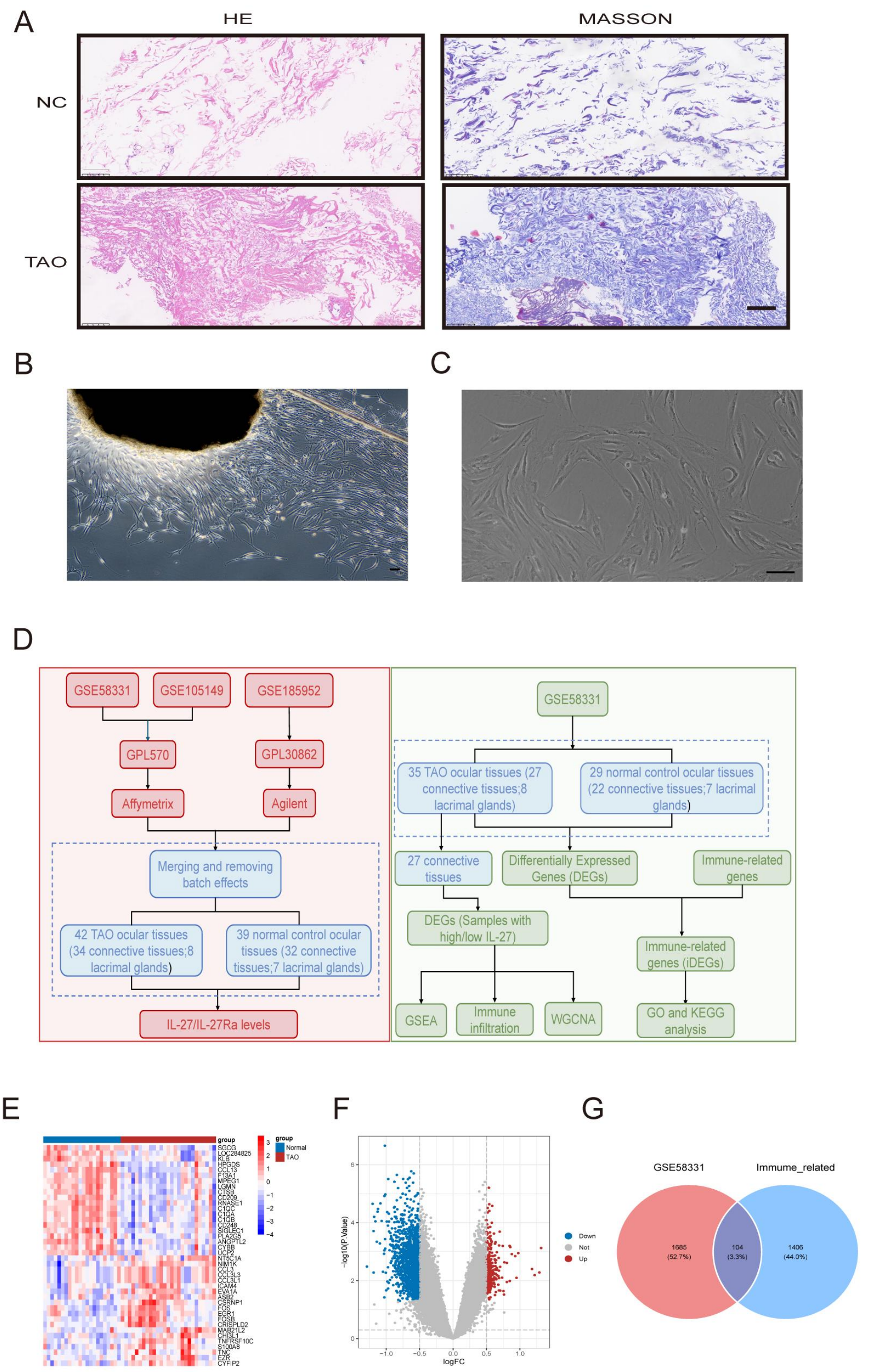

Fig.S2 Increased IL-27α/IL-27Ra in orbital tissue and OFs from TAO; related to Fig.2. (A) HE and MASSON staining for orbital connective tissues from TAO. (B-C) In vitro primary culture of human OFs. (D) Workflow chart of bioinformatic analysis. (E-F) Heatmap and Venn diagram of DEGs in GSE58331. (G) Establishment of iDEGs. Scale bars, 100 μm.

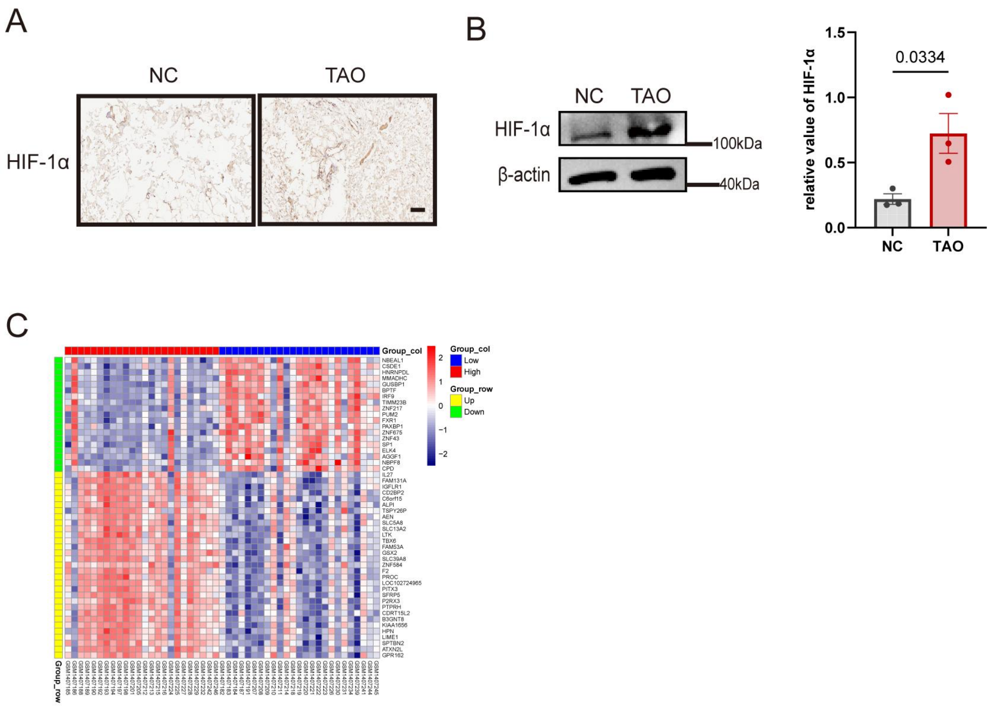

Fig.S3 The existence of hypoxic microenvironment in TAO; related to Fig.3. (A) IHC staining of HIF-1α in the orbital connective tissue from TAO and NC. (B) Immunoblot analysis of the indicated proteins in OFs from TAO and NC (n = 3). (C) Heatmap of DEGs based on expression of IL-27. Data are presented as means ± SEM; Each data point represents an individual experiment; unpaired two-tailed t test (B). Scale bars, 100 μm.

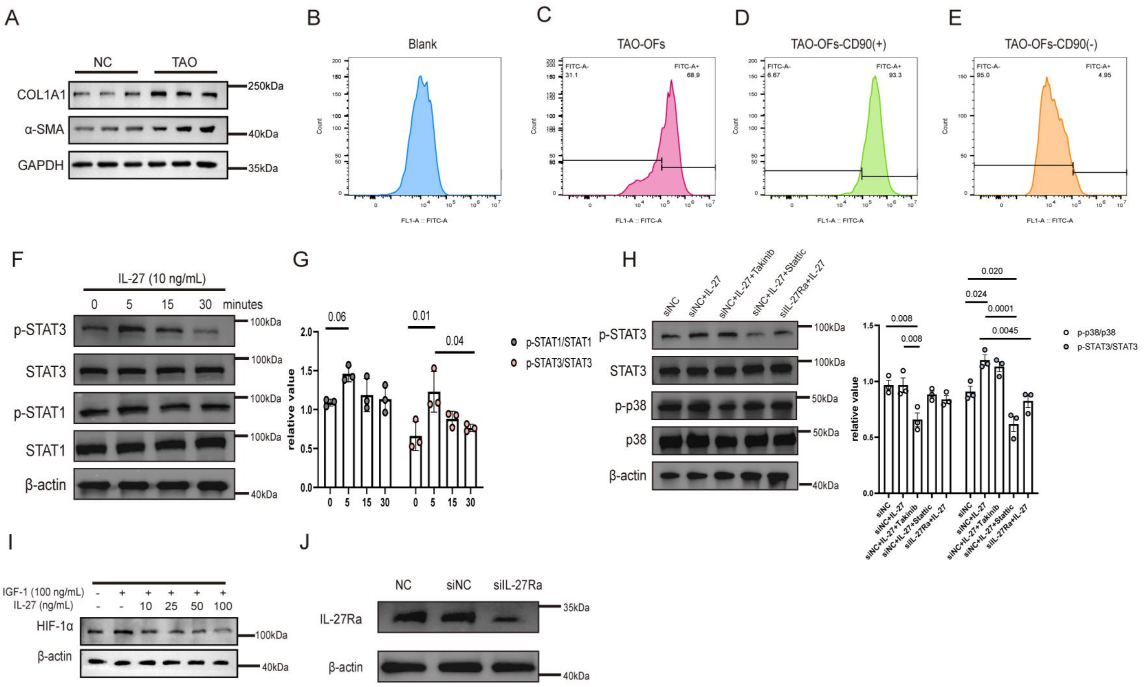

Fig.S4 IL-27 suppressed adipogenic differentiation in TAO-OFs; related to Fig.4 and Fig.5.

(A) Immunoblot of fibrosis markers. (B-E) Flow cytometry analysis was performed to assess CD90 expression in OFs, with panel (B) representing the negative control and panel (C) showing parental OFs. (D) The CD90+ subset was isolated from parental OFs after two rounds of flow cytometric sorting. (E) Similarly, the CD90- subset was isolated from parental OFs following two rounds of sorting. (F-G) Immunoblot analysis was conducted for the indicated proteins. (H) Immunoblot analysis of the selected proteins. (I) Immunoblot of HIF-1α under treatment of IL-27 and IGF-1.(J) Validation of siRNA knockdown efficiency.

Table S1 The baseline characteristics of disease controls.

Characteristics	UV	OC	GD
Sex, m/f	25/18	11/19	17/38
Age, y	46.86±15.17	47.50±18.30	45.00±14.46
WBC, 10^9/L	6.828±1.791	7.485 (5.890-9.473)	6.348±1.955

Abbreviations: WBC, white blood cell; UV, uveitis; OC, orbital cellulitis; GD, Graves' disease; m, male; f, female; y, year.
